# Validation of the Bangla PHQ-4 in rural and urban community samples from Bangladesh

**DOI:** 10.1371/journal.pone.0352459

**Published:** 2026-06-26

**Authors:** Muhammad Kamruzzaman Mozumder, Ummay Kulsum Keya, Fayaza Ahmed, Imtiazuddin Gazi, Konica Gop, Pooja Bhattacharjee, Md. Akramul Islam, Shayla Islam

**Affiliations:** 1 Department of Clinical Psychology, University of Dhaka, Dhaka, Bangladesh; 2 Center for Student Mental Health and Well-being, Barishal University, Barishal, Bangladesh; 3 BRAC Health Programme, BRAC, Dhaka, Bangladesh; University of São Paulo, BRAZIL

## Abstract

**Background:**

Shortage of qualified mental health professionals and limited access to specialized care, coupled with a high burden of mental health problems, particularly anxiety and depression, is a growing public health concern in Bangladesh. A possible solution being tested is the utilization of existing community resources. Availability of a psychometrically valid and reliable screening tool for early detection of mental health problems is of paramount importance. The present study aimed at testing the psychometric properties of the Bangla version of the ultra-brief PHQ-4.

**Methods:**

Employing practicing mental health professionals as data collectors, this cross-sectional study used data from 357 adults from an urban and two rural communities of Bangladesh.

**Results:**

Confirmatory factor analysis indicated excellent model fit for the two-factor structure of the Bangla PHQ-4 (*χ*^2^ = 0.103 at *p* > .01, RMSEA = 0.000, CFI = 1.000, TLI = 1.007, and SRMR = .006) for the total sample. The model demonstrated factorial invariance for sex, age, and mental health state. The scale demonstrated high internal consistency (McDonald’s ω = .869) and strong construct validity, evidenced by high correlation with the SRQ-20 (r = .747), K-6 (r = .820), self-rated mental health state (r = −.677), and interviewer-rated mental health state (r = −.680). The scale also demonstrates excellent diagnostic accuracy (AUC = .906), and acceptable sensitivity (82%) and specificity (83%) using a cutoff score ≥ 6.

**Conclusion:**

This study provided evidence of satisfactory psychometric properties of the Bangla PHQ-4 in terms of factor structure, group level invariance, internal consistency, and construct validity in Bangladeshi rural and urban community samples.

## Introduction

Early detection and intervention play an important role in intervening mental health problems [1]. With only 1169 qualified mental health professionals [2], the possibility of helping the huge population in need (18.7% adults and 12.6% children) and addressing the 92% treatment gap [3] remains slim for Bangladesh. A community-based approach to mental health using paraprofessionals linked with a referral network can be a feasible option for addressing this massive gap [4]. Due to the heavy workload and existing responsibilities of the healthcare service providers working in Bangladesh [5], it is understandable that using lengthy tools for the identification of mental health conditions in the community will be challenging for them. To address this issue, introducing a concise, easy-to-use screening tool would be highly beneficial. A brief tool for screening mental health problems in the community can be extremely useful in supporting these community-based service providers. The present study was therefore conceived to test the usability of the PHQ-4 in the Bangladeshi community sample.

Across the globe, depression and anxiety account for a major portion of the mental health disease burden [6,7] and are in need of recognition as a serious public health concern [8,9]. High prevalences of depression (39.8%) and anxiety disorders (26.1%) reflect the same concern for Bangladesh [9]. Thus, addressing depression and anxiety alone can possibly reduce at least two-thirds of the mental health disease burden in Bangladesh. Additionally, ease of identification and efficacy of intervention for these two conditions made these ideal candidates for a large-scale, cost-effective solution to address the mental health gap, which has been effectively proven with the experience of Improving Access to Psychological Therapies (IAPT) model [10].

While diagnostic clinical interviews offer precision in screening, the requirement for prerequisite skills and knowledge, as well as their time-consuming nature, make them impractical for large-scale population-level screening. Screening tools can facilitate wider use and early identification, especially in the low-resource context of Bangladesh. Assessment of depression and anxiety using comprehensive tools, such as the locally developed Anxiety Scale [11] and the Zahir Depression Scale [12] or translated tools such as the Bangla Beck Depression Inventory [BDI-II, 13] takes time and requires professional expertise for administration and interpretation. Therefore, these are unsuitable for large-scale community-level screening. The Depression Anxiety and Stress Scale [14], Hospital Anxiety and Depression Scale [HADS; 15], or the General Health Questionnaire [GHQ-12; 16] held a better promise in this regard. However, negative feedbacks (e.g., difficult wording, complexities of administering, time required) from community-level service providers make the Bangla translated version of these tools unappealing. Brief tools such as the Patient Health Questionnaire – 9 [PHQ-9; 17] and the Generalized Anxiety Disorder-7 [GAD-7; 18], with their acceptable reliability, validity, and diagnostic performances, are widely used for detecting depression and anxiety across the globe [19,20]. However, the need for an even shorter tool was always there. The ultra-brief 4-item Patient Health Questionnaire [PHQ-4; 21] was developed in this regard by combining two items from the PHQ-9 and two other items from the GAD-7 to assess depression and anxiety.

For its brevity, ease of administration, and superior psychometric properties, the PHQ-4 has gained wide acceptance, leading to its translation and use in many languages, cultural groups, and countries, including Spanish in Columbia [22], Quechua in Peru [23], Korean in South Korea [24], Mandarin in China [25], Malay in Malaysia [26], Arabic in Lebanon [27], German in Germany [28], Greek in Greece [29], Swahili in Tanzania [30], and Japanese in Japan [31]. The PHQ-4 has also been validated on different population groups, including patients with health [32] and mental health [24] conditions, adolescents [30], students [33], and pregnant women [34]. Systematic review of studies conducted on clinical and nonclinical samples across 19 countries indicates adequate psychometric properties (Cronbach’s alpha = .72 −.88, convergent validity r = .44 −.83) of the PHQ-4 [35]. Generally, the two-factor model of the PHQ-4 has been found to fit better with community samples [36,37], while the one-factor model indicated better fits with clinical samples [24]. The systematic review mentioned earlier also indicated adequate fit for the two-factor model of the PHQ-4, with CFI ranging from 0.97 to 1.00, TLI from 0.90 to 1.00, and RMSEA from 0 to.14 [35].

Both the PHQ-9 and the GAD-7 have been translated and validated in Bangladesh [38,39], which implies that the four items taken from these two instruments (i.e., the PHQ-4) have already gone through some forms of field testing. The PHQ-4 and its two constituents, i.e., PHQ-2 and GAD-2, have already been used as research tools in Bangladesh [40–42], though they have not yet undergone psychometric testing. Therefore, the present study aimed to assess the psychometric properties, i.e., factor structure, reliability, and validity of the Bangla PHQ-4 in Bangladeshi community sample.

## Methods

### Participants

Three hundred and fifty-nine individuals from two rural (Netrokona and Khulna) and one urban (Dhaka) community of Bangladesh participated in this study. Missing values in two items resulted in the removal of two individuals, reducing the sample size of the present study to 357. The remaining data still contained some missing values in other variables (n = 11 for self-rating of mental health state, n = 12 for Interviewer’s Rating of mental health state, n = 21 for diagnosis by interviewers, and n = 4 for the SRQ-20 score), which were only 0.8% of total data values spread across 9.5% of cases. However, these were retained as they did not interfere with the analysis. Little’s MCAR test indicates no patterns in the missingness (*χ*^2^ = 11.535, p > .05) and therefore, no other case was deleted or no imputation was carried out. A cross-sectional selection of participants targeting maximum variation in sociodemographic characteristics was ensured during data collection (Table 1). The demographics indicate a higher proportion of females (57%) and married participants (78%), with the majority (36%) reporting family as the source of income. The participants’ ages of ranged from 18 to 80 years (*M* = 38.59, *SD* = 13.08).

**Table 1:** Sample characteristics.

	Total Sample (n = 357)	Rural Sample (n = 251)	Dhaka (Urban) (n = 106)	Khulna (Rural) (n = 158)	Netrokona (Rural) (n = 93)
Gender					
Male	152 (42.6)	100 (39.8)	52 (49.1)	62 (39.2)	38 (40.9)
Female	205 (57.4)	151 (60.2)	54 (50.9)	96 (60.8)	55 (59.1)
Age					
Young Adult (18–35 yr)	164 (45.9)	112 (44.6)	52 (49.1)	69 (43.7)	43 (46.2)
Middle-Aged (36–55 yr)	150 (42.0)	104 (41.4)	46 (43.4)	69 (43.7)	35 (37.6)
Older Adult (56 + yr)	43 (12.0)	35 (13.9)	8 (7.5)	20 (12.7)	15 (16.1)
Educational level					
Illiterate	46 (12.9)	39 (15.5)	7 (6.6)	14 (8.9)	25 (26.9)
Up to primary	84 (23.5)	76 (30.3)	8 (7.5)	38 (24.1)	38 (40.9)
Up to SSC	83 (23.2)	73 (29.1)	10 (9.4)	60 (38)	13 (14)
Up to HSC	37 (10.4)	30 (12)	7 (6.6)	20 (12.7)	10 (10.8)
Up to graduation	42 (11.8)	21 (8.4)	21 (19.8)	16 (10.1)	5 (5.4)
Up to post-graduation	65 (18.2)	12 (4.8)	53 (50)	10 (6.3)	2 (2.2)
Source of Income					
Job	89 (24.9)	37 (14.7)	52 (49.1)	23 (14.6)	14 (15.1)
Business	52 (14.6)	40 (15.9)	12 (11.3)	24 (15.2)	16 (17.2)
Family	128 (35.9)	101 (40.2)	27 (25.5)	71 (44.9)	30 (32.3)
Physical labor or others	88 (24.6)	73 (29.1)	15 (14.2)	40 (25.3)	33 (35.5)
Marital Status					
Unmarried	43 (12)	17 (6.8)	26 (24.5)	10 (6.3)	7 (7.5)
Married	278 (77.9)	216 (86.1)	62 (58.5)	140 (88.6)	76 (81.7)
Others – Separated, Divorced, Widow(er)	36 (10.1)	18 (7.2)	18 (17.0)	8 (5.1)	10 (10.8)
Presence of MH* Condition					
Yes	71 (19.9)	46 (18.3)	25 (23.6)	33 (20.9)	13 (14)
No	264 (73.9)	184 (73.3)	80 (75.5)	120 (75.9)	64 (68.8)

Note: the percentages in parentheses may not sum up to 100 due to missing values in the data; * MH = Mental Health.

### Instruments

The 4-item Patient Health Questionnaire [PHQ-4; 21]. The PHQ-4 is a short composite anxiety and depression scale designed by amalgamating the 2-item anxiety screener (GAD-2) and the 2-item depression screener (PHQ-2). As understandable from its source construction, the PHQ-4 demonstrates a two-factor structure, namely anxiety and depression, with high factor loadings of the relevant items to the specific factors [21]. The instrument demonstrates acceptable reliability, construct validity (strong association with functional impairment indicators), and good measurement invariance across culture groups, age, and gender [21,28,43].

The Self-Reporting Questionnaire [SRQ-20; 44]. As a screener, the PHQ-4 is likely to have strong concordance with the SRQ-20 [44]; therefore, it was used to measure construct validity of the PHQ-4. The SRQ-20 is a widely used screening tool that measures overall psychological morbidity in the general population worldwide [45,46]. This 20-item instrument with dichotomous (no-yes) scaling has been reported to be a moderately valid and reliable tool for mental health screening in Bangladesh [47]. Worldwide estimates suggest a score ≥ 8 as the suitable cutoff value with acceptable sensitivity (81%−90%) and specificity (58%−95.2%) in screening people for psychological morbidity across populations from different countries [see 44]. However, analysis from the urban community sample indicated a score ≥ 7 as the most suitable cutoff value for Bangladesh [47].

Kessler Psychological Distress Scale [K-6; 48]. Psychological distress is closely connected with mental health. The six-item K-6 was therefore used to assess the construct validity of PHQ-4. With a score ranging from 0 to 24, a higher score indicates a greater distress in the K-6. Results from meta-analysis indicated excellent internal consistency (mean Cronbach’s alpha = .84, 95% CI [.80 −.88]) of the K-6 [49]. Bangla version of the K-6 has also been reported to have adequate psychometric properties (internal consistency alpha = .87, test-retest reliability r = .80, validity with depression r = .68) [50].

Mental Health State. The mental health state of the respondents was measured using two indices: i) a self-rating by the respondents, and ii) rating by the interviewer. Both indices used an 11-point (0–10) rating scale, where 0 indicated the worst mental health state and 10 indicated the best mental health state. These rating scales were used to test the construct validity of the Bangla PHQ-4 in a convergent manner. Additionally, the interviewers, all of who were practicing mental health professionals, assessed the respondents for mental health state using their clinical judgment. As the goal was not to diagnose specific mental disorders, they did not use a structured diagnostic interview. Rather, they used clinical interviews to reach a generic conclusion about the participant’s caseness.

### Procedure

The data used in this study were collected between 20 October and 24 November 2025 by six trained mental health professionals with academic backgrounds in clinical psychology and psychology. They administered the questionnaire through face-to-face interviews. As all the data collectors were practicing clinicians, they relied on clinical interviews in detecting the presence of mental health conditions among the participants. Due to contextual realities (e.g., variations across community structures and resource availability), the team did not use a standardized printed protocol for participant selection. However, they followed pre-planned procedures, agreed upon and updated daily in team meetings, with feedback on the completion of data collection for each day.

The data collectors walked through the community areas (such as residential areas and marketplaces) of the selected study sites and invited the individuals they encountered to participate in this study. For interviews in the households, each team member was assigned to a specific area in a community. For interviews in community gathering places (e.g., a marketplace), multiple interviewers worked at the same location, and it was ensured that no participant was interviewed twice. Irrespective of the location, the interviewers ensured a suitable place that was comfortable for participants to respond. No name or identification details of the participants were collected. Participation was voluntary based on their verbal consent upon receiving details on the nature of the study. Each questionnaire survey took about 15–18 minutes to complete; however, for those who appeared to have a mental health condition, the inclusion of a clinical interview increased the length considerably. The refusal rate among all participants approached was 12.26%, indicating a good response rate among the community respondents [51]. The interviewers accepted all refusals without asking for any reason or further requesting participation. Verbal consent was preferred and used instead of written consent due to sensitivity around signing documents, which is generally observed among the community participants in Bangladesh [52]. Ethical approval for the study was obtained from the Department of Clinical Psychology ethics committee (Project # IR250801; date of approval 07 September 2025).

### Data Analysis

JASP [53] and SPSS [54] were used to analyse the data in this study. The weighted least squares with mean and variance adjustment (WLSMV) estimation method was used in the confirmatory factor analysis (CFA) to test the goodness-of-fit of the widely reported two-factor model of the PHQ-4. Being the most commonly suggested indices, the chi-square (*χ*^2^), the ratio of chi-square to *df* (*χ*^2^/df), the root mean square error of approximation (RMSEA), the comparative fit index (CFI), the Tucker-Lewis index (TLI), and the standardized root mean square residual (SRMR) were used to test the adequacy of model fit. The criteria used for model fit using these indices were *χ*^2^ significant at *p* ≥ .01, RMSEA ≤ .06, CFI ≥ .95, TLI ≥ .95, and SRMR ≤ .08 [55,56]. Testing for measurement invariance was carried out to assess the comparability of factor structure across groups, as needed for meaningful comparisons in future research using the Bangla PHQ-4. Four models of invariance were tested, with relevant constraints placed across the groups. These were configural invariance (constraining the factor structure), metric invariance (constraining the factor loadings), scalar invariance (constraining all item intercepts), and strict invariance (constraining residual variances). Change in CFI (ΔCFI) ≤.01, change in SRMR (ΔSRMR) ≤.03, and non-significant change in chi-square (Δ*χ*^2^) at p < .01 were used as the criteria for indication of measurement invariance [57].

Internal consistency reliability was assessed using McDonald’s omega instead of the more popular Cronbach’s alpha because of its robustness. The construct validity of the Bangla PHQ-4 was assessed using three convergent methods, which were correlations with the SRQ20, self-ratings of mental health (MH) state, and interviewers’ ratings of mental health (MH) state.

### Findings

To understand the socio demographic distribution of participants from rural and urban locations, Chi-square tests were carried out. The result indicates no significant difference in distribution of sex (χ^2^ = 2.589, df = 1, p > .05) and age (χ^2^ = 2.919, df = 2, p > .05) of the participants across locations. However, significant difference in distribution was observed for marital status (χ^2^ = 33.890, df = 2, p < .01) and source of income (χ2 = 47.567, df = 3, p < .01). Married participants overly represented the rural sample (86.1%) while for urban sample the distribution was slightly better (24.5% unmarried, 58.5% married, 17% others). Job was the most prevalent (49.1%) source of income in Urban, while for Rural it was family (40.2%).

The results of psychometric analysis of the PHQ-4 are presented in four sub-sections discussing confirmatory factor analysis, internal consistency reliability, construct validity, and diagnostic performance of the Bangla version of the tool.

### Confirmatory factor analysis (CFA)

**Fig 1:**
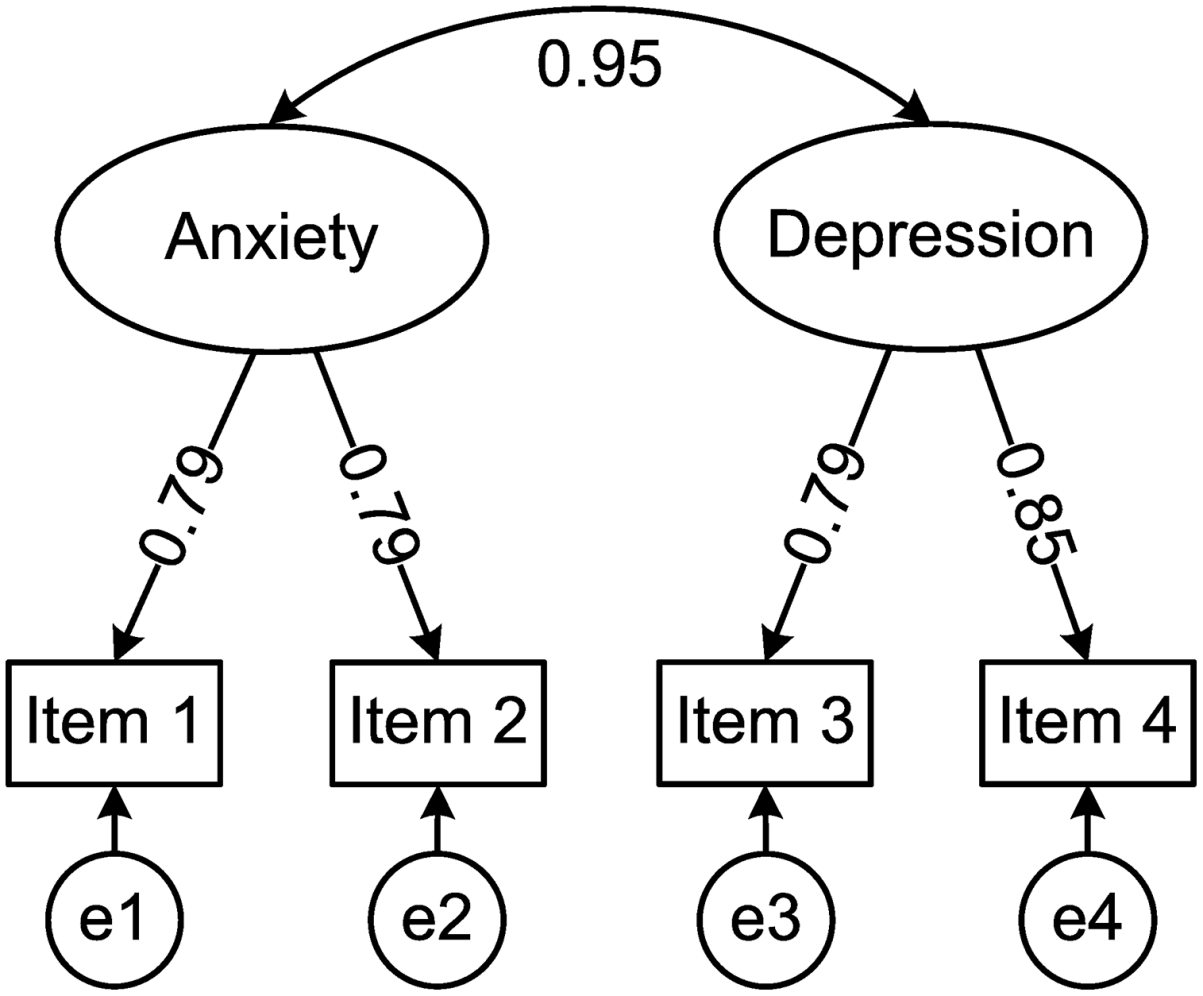
CFA of the two-factor model of the Bangla PHQ-4 on the community sample. Error terms for the respective items are represented as e1- e4.

The data indicate excellent model fit across multiple indices (χ^2^ = 0.103 at p = .748, RMSEA = 0.000 (CI: 0.000–0.097), CFI = 1.000, TLI = 1.007, and SRMR = .006) for the widely reported two-factor model of the PHQ-4 (see Fig 1).

Multigroup CFA demonstrated that the two-factor model of the Bangla PHQ-4 is invariant across sex, age, and state of mental health condition (Table 2). All the Δχ² were non-significant, and both ΔCFIs as well as ΔSRMRs met the fit criteria. For location, the model was reported to be not admissible due to the covariance matrix of latent variables not being positive definite. Therefore, measurement invariance tests could not be computed for location.

**Table 2:** Measurement invariance of the Bangla PHQ-4 across groups.

Groups	Invariance Test	Model Fit Indices					Changes in Fit Indices		
		χ²	df	p	CFI	SRMR	Δχ²	ΔCFI	ΔSRMR
Sex (Male, Female)	Configural	0.222	2	.895	1.000	.006	–	–	–
	Metric	1.488	4	.829	1.000	.022	1.266	0.000	.016
	Scaler	2.697	6	.846	1.000	.024	1.209	0.000	.002
	Strict	4.515	10	.921	1.000	.031	1.818	0.000	.007
Age (Young Adult, Middle-Aged, Older Adult)	Configural	0.192	3	.979	1.000	.008	–	–	–
	Metric	2.148	7	.951	1.000	.019	1.956	0.000	.011
	Scaler	4.659	11	.947	1.000	.026	2.511	0.000	.007
	Strict	9.164	19	.971	1.000	.043	4.505	0.000	.017
Mental Health Condition (Yes, No)	Configural	0.591	2	.744	1.000	.007	–	–	–
	Metric	0.837	4	.933	1.000	.012	0.246	0.000	.005
	Scaler	4.717	6	.581	1.000	.031	3.880	0.000	.019
	Strict	12.734	10	.239	0.991	.061	8.017	0.009	.030

### Internal consistency reliability

McDonald’s omega (ω = .869) indicates acceptable internal consistency reliability of the Bangla PHQ-4 (Table 3). The two subscales also demonstrate strong interrelation (r = .746), further supporting the internal consistency of the PHQ-4. Sufficient inter-item correlation was observed for items within the anxiety subscale (*r* = .626, *p* < .01) and the depression subscale (*r* = .668, *p* < .01).

**Table 3:** Reliability and validity of the Bangla PHQ-4.

	Total Sample (n = 357)	Rural Sample (n = 251)	Dhaka (Urban) (n = 106)	Khulna (Rural) (n = 158)	Netrokona (Rural) (n = 93)
Internal consistency reliability					
Coefficient ω	.869	.860	.888	.882	.831
Construct validity					
SRQ20	.747**	.739**	.767**	.762**	.703**
K6	.820**	.795**	.880**	.855**	.698**
Self-rating of MH state*	−.677**	−.666**	−.732**	−.694**	−.618**
Interviewer’s rating of MH state*	−.680**	−.659**	−.724**	−.660**	−.658**

* MH = Mental Health; Rating on a scale of 0 (poor) to 10 (perfect); ** significant at p < .01

### Construct validity

Table 3 indicates strong correlations between the PHQ-4 and the SRQ-20 (*r* = *.747*, *p* < .01) as well as PHQ-4 and the K-6 (*r* = *.820*, *p* < .01). The two subscales also demonstrate strong significant correlations with the SRQ-20 (r = .676 for anxiety and r = .719 for depression). High correlations between scores on the PHQ-4 and respondents’ self-rating of mental health (r = −.677) as well as interviewers’ rating of the respondents’ mental health state (r = −.680) also demonstrate construct validity of the Bangla PHQ-4.

### Diagnostic performance

**Fig 2:**
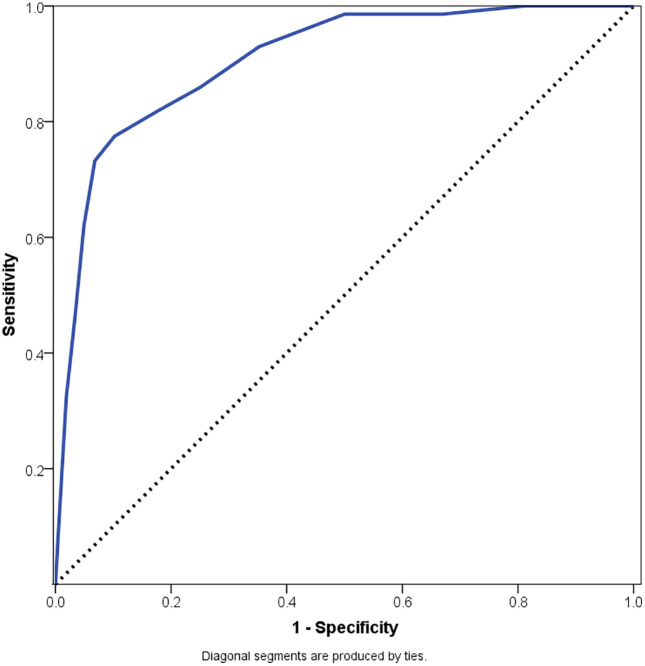
Receiver operating characteristic curve for the Bangla PHQ4.

The receiver operating characteristic curve indicated (AUC = .906) good diagnostic performance of the Bangla PHQ-4 using diagnoses made by the data collectors who were also active clinicians involved in practicing psychological therapy (Fig 2). They identified 71 participants who have a mental health condition needing psychological support. Sensitivity and specificity of the scale with different cutoff values are presented in Table 4.

**Table 4:** Sensitivity and specificity of the Bangla PHQ4 scale with different cutoff scores.

Cutoff score	Sensitivity	Specificity
1	100	19
2	99	33
3	99	50
4	93	65
5	86	75
6	82	83
7	77	90
8	73	93
9	62	95
10	46	97
11	32	98
12	20	99

* Suitable cutoff for best combination of sensitivity and specificity.

Sensitivity-Specificity calculations for the full range of scores of the Bangla PHQ-4 is presented in Table 4. While Youden’s and other indices [see [58]] suggest score ≥ 7 as the cutoff with disproportionate sensitivity and specificity (77 and 90), a cutoff score ≥ 6, on the other hand, provides sensitivity-specificity with the least difference (82 and 83) between the two, making it a possible candidate for the optimal cutoff value with the best combination of sensitivity and specificity (Table 4).

## Discussion

In the context of the need for a suitable and brief tool for screening mental health conditions at the community level, this study evaluated the psychometric properties of the Bangla PHQ-4. Despite a few instances of the PHQ-2 and GAD-2 being used for research purposes, the psychometric properties of the Bangla PHQ-4 had never been tested before in a community sample of Bangladesh. Drawing on samples from two rural and one urban communities in Bangladesh, analyses were carried out to test the factor structure, internal consistency, construct validity, and diagnostic performance of the Bangla PHQ-4.

Consistent with the studies from different other contexts, the Bangla PHQ-4 confirmed the widely reported two-factor structure of the PHQ-4 [25,27,59]. Goodness of fit was indicated by all six fit indices across the total sample. The fit indices for the Bangla PHQ-4 (CFI = 1.000, TLI = 1.007, RMSEA = 0.000) are consistent with the validation studies carried out in Southeast Asia (CFI = 1.000, TLI = 1.000, RMSEA = 0.000) [59] and in other countries as reported in a systematic review on findings from 19 countries [35]. In multigroup CFA for sex, age, and mental health condition, the two-factor model demonstrated excellent fit across all four invariance tests (configural, metric, scalar, and strict) with increasing level of model constraints. This supports the robustness of the Bangla PHQ-4 in assessing anxiety and depression across sex, age, and mental health state. Similar to the present findings, studies commonly reported invariance of the PHQ-4 for sex, age, and location [59,60]. However, in the present study, invariance testing could not be carried out for location because the covariance matrix of latent variables was not positive definite.

Published literature from across the globe indicates the PHQ-4 as an internally consistent instrument. McDonald’s omega was used as a robust indicator of internal consistency for the Bangla PHQ-4, which (ω = .869) indicates superb internal consistency, especially notable for an ultra-brief 4-item tool. Findings from China [omega = .916; 61], Japan [alpha = .84; 31], Korea [alpha = .792; 24], and those reported in a systematic review [alpha ranging from.72 to.88; 35] indicate internal consistency of different language versions to be comparable with that of the Bangla PHQ-4. Subsequent analyses revealed good inter-item correlation between the pair of items in the anxiety (*r* = .626) and depression (*r* = .668) subscales. The two subscales also show a strong correlation (r = .746). All these indicate sufficient internal consistency of the Bangla PHQ-4 and prove its comparability to the global evidence on other versions, including the English [21], Arabic [27], Korean [24], Japanese [31], Chinese [25], and German [28] versions of the PHQ-4.

Construct validity of the PHQ-4 has been tested and published with a wide range of constructs, including loneliness [36], self-esteem [37], resilience [37], life satisfaction [37], quality of life [21,32], depression [24,27], anxiety [24,27], and stress [27,59]. The choice of selecting SRQ-20 for testing construct validity of the Bangla PHQ-4 was based on their commonality in utility (screening of mental health conditions). Though it may not be very precise, the self-rating of mental health state is a simple, easy-to-understand measure and probably the easiest option for assessing mental state. The findings demonstrate strong correlations of the PHQ-4 with the SRQ-20, the K-6, self-rating of mental health state, and clinician’s rating of mental health state (r = .747,.820, −.677, & −.680).

As the search for a suitable tool to screen mental health at the community level was the prime goal for conducting this study, the diagnostic performance of the Bangla PHQ-4 was assessed using diagnostic screening done by practicing mental health service providers (who also served as the data collectors for the study). The findings indicate a better diagnostic performance (AUC = .906) of the Bangla PHQ-4 compared to other versions of the PHQ-4 [33,62]. This may be linked with the use of clinicians in data collection and their use of clinical interviews in case detection for the present study.

Youden’s index or other established methods [see 58] suggest 7 as the cutoff value with disproportionate sensitivity and specificity (77 and 90). Instead, we used the “least difference method” considering the tool’s utility in both directions. In this case, the use of 6 as the cutoff value yielded a more balanced estimate of sensitivity and specificity (82 and 83). Therefore, a cutoff score of ≥ 6 can be considered optimal for mental health screening at the community level. A lower cutoff (< 2) will yield the highest sensitivity (100), sacrificing the specificity of the scale. On the other hand, a higher cutoff score (>11) will yield the highest specificity (99) while sacrificing sensitivity of the scale. The sensitivity and specificity table would help any user choose a suitable cutoff value in accordance with their purpose.

The excellent psychometric properties of the Bangla PHQ – 4 demonstrated in this study establish this as a highly potent screening tool for Bangladesh. The understandability of the items, shorter length, and ease of administration are likely to increase its use in resource-constrained communities, primary healthcare settings (e.g., community clinic), or in disaster response. It can also be useful for non-specialist healthcare professionals for screening and subsequently making appropriate referrals to the mental health service. With the evidence of robustness demonstrated in group-level invariance tests, the Bangla PHQ – 4 can be especially useful as a brief research tool for epidemiological and comparative studies on mental health states across different population groups.

### Strengths and limitations

The involvement of mental health practitioners as data collectors was a unique strength of the present study. Their involvement in the scale administration and identification of mental health conditions ensured valid and acceptable diagnostic screening. The prevalence of mental health conditions, as identified by the data collectors, was similar to the national mental health survey findings [9], providing further validation regarding the accuracy of the screening done by the data collectors. The data were collected from both rural and urban communities, employing convenience sampling, while also ensuring maximum variability of age, sex, socio-economic status, and marital status, thus ensuring wider representation. Compared to the acceptable or average response rates reported in survey research [51,63], the high response rate (88%) in the present study is an additional strength, indicating the reduced risk of bias and high representativeness of the data.

Primarily because of door-to-door interviews in the community, this study could not ensure proportional data collection, leading to disparities in certain demographic variables such as, married (77.9%), females (57.4%), and dependent on family income (35.9%), resulting in over-representation by these groups. The majority of data was collected during working hours, which might have reduced access to the working population. Moreover, the occurrence of an earthquake during the latter period of data collection might have some unavoidable influence on some of the data collected from the urban population.

The present study does not reflect how the scale would perform in inpatient settings. Utilization of clinical samples from the hospital could be useful in ascertaining a more accurate estimate of diagnostic performance and sensitivity-specificity of the Bangla PHQ-4. Future research can consider this along with the utilization of a larger random sample, an increased range of time for data collection, assessment of stability reliability, and further testing of the construct validity of the Bangla PHQ-4.

## Conclusions

Despite the huge need for a short yet reliable, valid, and robust tool for screening mental health conditions in Bangladesh, the PHQ-4, an ultra-brief screening tool widely used across the globe, has never been validated in Bangladesh. The present study was therefore carried out to assess the psychometric properties of the Bangla PHQ–4 in rural and urban communities in Bangladesh. Active clinicians collected data from the community through door-to-door survey using a cross-sectional design and maximum variation sampling.

Findings demonstrate the Bangla PHQ-4 as a psychometrically sound instrument with comparable, and in some cases, superior performance compared to the other versions of the PHQ-4 tested across the globe. The robustness of the tool across rural and urban communities of Bangladesh, as demonstrated by the same factor structure with strong model fit indicated in all indices, suggests its suitability for use in both rural and urban populations.

With shorter length and ease of use, the Bangla PHQ-4 is expected to aid the community-level service providers as well as the researchers in fast and accurate screening of depression and anxiety. The estimates of sensitivity and specificity of the Bangla PHQ-4 provided in this study are likely to be specifically useful in this regard.
