# Supplementary material for: Validation of the Bangla PHQ-4 in rural and urban community samples from Bangladesh
Source: PLoS One. 2026 Jun 26;21(6):e0352459. doi: 10.1371/journal.pone.0352459 (PMC13308809; doi:10.1371/journal.pone.0352459)
Supplement: Attachment — Submitted filename: Response to Reviewers.docx [file pone.0352459.s002.docx]

**Response to Review Comments to the Author**

**Reviewer #1:**

Dear authors, Thank you for your efforts. The idea is good and necessary for the country and native language, however, the paper needs a lot of work. Here are my comments:

*Thank you for your support. We truly appreciate the contribution of the reviewers in gatekeeping knowledge. We have tried our best to address your comments.*

-The paper needs to be corrected by a professional English native speaker. The sentences structure is weak.

***Response:***

*We understand that reading English-written work by a non-native speaker can be uncomfortable, and we sincerely apologise for that. Unfortunately, none of the team members was a native speaker. After your comment, we invited a British-born graduate student, a British-born post-graduate student, and a British-born Medical Doctor to review the work, and all of them cleared it for any lack of understanding or major grammatical errors. Nonetheless, we have revised some linguistic and grammatical issues as well as typos to improve the readability of the work.*

-The introduction needs to be reshuffled as follows:
start with a general introduction about mental health status in the world and in the country.
state all scales that are used to assess depression and anxiety and state which ones are validated in Bangladesh
then you should mention why you want to validate the PHQ-4 scale.
State the languages PHQ-4 is validated in
Rationale for validating the scale in Bangladesh
Objectives and hypotheses

***Response:***

*We preferred to present an opening paragraph at the beginning to introduce the idea to the readers. However, from the second paragraph onward, we gradually funnel down toward the objective (the team preferred to phrase this as ‘aim’ instead of ‘objective’). Moreover, we have made a few revisions as per your suggestion. Details on the locally available tools for assessing depression and anxiety have been added (line 64-73). We have also added languages in which the PHQ-4 has been validated (line 82-85). Additionally, we have added details on validation findings from published literature (line 108-110; line 112-114).*

Regarding the methods:
-Table 1 contains a low number of participants for certain categories. These categories should be merged together to have a decent number for the analysis.

***Response:***

*We have merged several subcategories under ‘marital status’ and ‘source of income’ as per your suggestion. We have also added a new categorisation of age.* (Table 1)

-The estimation method should be WLSMV since the data is ordinal. Can the authors explain why they used the maximum likelihood? Otherwise, please redo the analysis using WLSMV.

***Response:***

*Thank you for this extremely useful suggestion. We have now re-analysed the data with the estimator specified as WLSMV, and have revised the result section accordingly. (line 183-184; line 218-219)*

-The fit indices should be obtained from the total sample.

***Response****:*

*As per your suggestion, we have kept only the fit indices for the total sample and described findings on fit indices in the text, removing the table. (line 218-219).*

-Measurement invariance should be done by gender, by region of living, education level, etc (as deemed necessary by the authors). The stated results are not fully accurate.

***Response***:

*We have carried out measurement invariance tests for sex, age, and mental health state and added the findings as Table 2. (line 225-232)*

-The authors did not use other scales to assess convergent, divergent, or criterion validity?

***Response****:*

*We already have a plan to conduct a larger study to test the construct validity (convergent, divergent), and test-retest reliability of the tool. However, as this is an already developed scale with proven criterion validity, we did not plan to conduct testing for criterion validity.*

-The PHQ-4 scale has an already established cutoff values. Why did the authors do a ROC curve analysis? Please explain.

***Response****:*

*As the cutoff value may differ in different cultural contexts, we had to assess diagnostic performance and cut-off value for the Bangla translated PHQ-4 before the cutoff value can be used in Bangladesh. It should be noted that this is going to be the first published study with cutoff value of PHQ-4 for the Bangladeshi sample.*

For the discussion, the results of the current study should be compared to the ones from other countries (fit indices, alpha and omega values, measurement invariance, etc.)

***Response****:*

*As per your suggestion, we have added a comparative discussion on the psychometric properties of the tool.* *(line 276- 286, line 290-293; and also line 108-110; line 112-114)*

-Authors should add a clinical implications paragraph to state how their results would benefit other healthcare professionals.

***Response****:*

*Thank you for this suggestion. We have added a brief paragraph addressing the implications of the findings. (line 324-332)*

-Did the authors do a test-retest?

***Response****:*

*We have not done test-retest of the tool. We are planning to conduct a larger study to test the construct validity and test-retest reliability of the tool.*

I hope you find these comments helpful. Good luck and Happy New Year 2026.

*Thank you for spending time reviewing this article. These comments were extremely useful for our team, not only in improving the current article but for our upcoming work as well.*

**Reviewer #2:**

First of all, thank you very much for the opportunity to review this manuscript. Overall, the paper is well structured, comprehensive, and addresses a highly valuable topic: the measurement of instruments in psychology. This area has wide-ranging applications and represents a meaningful contribution to scientific knowledge.
With the aim of strengthening the manuscript, I would like to offer the following comments and suggestions:

*Thank you for your support. We truly appreciate the contribution of the reviewers in gatekeeping knowledge.*

Please consider citing the following article: https://www.nature.com/articles/s41598-023-41560-0

***Response****:*

*We have already cited similar and more recent study findings. We were not sure how citing this would add value to the current article. Therefore, we did not consider citing the suggested article.*

Line 91: Please specify the initial and final sample sizes, as well as any missing data. The current wording in this line raises some uncertainty.

***Response****:*

*Thank you for this useful feedback. We have the total number of participants and added the necessary details on missing value. (line 103, line 106-111)*

Table 1: Please indicate (e.g. a chi-square test or t-test) whether there are statistically significant differences in sociodemographic characteristics such as sex and age according to place of origin.

***Response:***

*We have added the analysis in the findings section.* (line 205-212.)

Line 114: Please verify whether the reference cited is correct.

***Response****:*

*Thank you for notifying this. We have changed this citation.* (line 133)

The sampling procedure raises several questions. Was a standardised protocol used when interacting with participants? If so, could you provide or publish the script or guidelines used? A more detailed description of the recruitment and approach would be beneficial.
Where were the interviews conducted (e.g., in the street)? How long did they last? It is understood that four instruments were administered: PHQ-4, SRQ-20, a self-rated mental health measure, and informed consent. It is also assumed that participants were informed about the study, its duration, and the consequences of declining or withdrawing, as well as whether they had previously participated in the study with another interviewer. Please clarify these aspects.

***Response****:*

*Thank you for these very useful comments. We have added some text in the procedure section to improve clarity of understanding as per your comments. (line 154-158, line 161-165, line 167-169, line 171-172, line 174-179)*

A subsection on statistical analysis is missing within the Methods section. I recommend incorporating the content currently presented in lines 147–155 into this subsection, with the Results section beginning at line 156.

Line 163 appears to describe methodological aspects. Please move this content to the statistical analysis subsection.

Similarly, the content in line 169 relates to statistical methodology and should be included in the appropriate subsection.

Content in line 179 should be relocated to the Methods section.

***Response****:*

*We thought that describing the data analysis method in line with the result would provide the readers with better connectivity. However, we agree with your suggestions and have added a data analysis section incorporating the indicated sentences along with a few connecting sentences.* (line 182- 203)

The term “MH” (presumably referring to mental health) is not defined in the manuscript. Please clarify its meaning.

***Response****:*

*Thank you for notifying this. Now we have added this to the text as well as under the table. (line # 119, 202-203, 241)*

Within the Methods section, it is not sufficiently clear that, following the interviews, the interviewers conducted a diagnostic assessment based on participants’ responses, classifying cases as either positive or negative for impaired mental health. This clinical classification appears to have been subsequently used for the AUC analysis. Please clarify this process.

***Response****:*

*To explore the presence of mental health conditions, the interviewer conducted an additional assessment based on the responses provided by the participants. However, as these interviews were not targeted to diagnose a specific disorder, the interviewers did not use SCID or other clinical diagnosis approaches for identifying a specific clinical disorder. As per your comment, details have been added to the instrument section. (line 144-148)*

Table 2: Please omit leading zeros before decimal points.

***Response****:*

*Thank you for noticing this. Based on the comments of the other reviewer, we have removed the older Table 2 and replaced it with a new one.*

*As per your comments, we have checked the manuscript and have omitted ‘0’s before the decimal point for p-values, SRMR values, alpha value and omega value. However, as per APA 7th recommendations, we have kept the ‘0’s before the decimal point intact for some other values which can go above ‘1’ .*

Please apply the “closest top-left” method to estimate the optimal cut-off point (alternatively, Youden’s index could also be considered). At present, the justification for selecting a cut-off point of 6 as optimal is not sufficiently supported.

***Response****:*

*We did not use Youden’s index or other established methods, as these indicate a cutoff with disproportionate sensitivity and specificity (77 and 90). Instead, we used a “least difference method” considering the usefulness of the tool in both directions. In this case, the cutoff 6 gave a more balanced estimate of sensitivity and specificity (82 and 83). However, as we have given the sensitivity and specificity table in the article, any user can choose a suitable cutoff value in accordance with their purpose.*

*Thanks to your comments, we have added these details to the manuscript for the readers. (line 257-261, line 315-318)*

Lines 217, 218, and 230: Please report values to three decimal places.

***Response****:*

*Revised as per the suggestion*

*Thank you for spending time reviewing this article. These comments were useful for our team, not only in improving the current article but for our upcoming work as well.*
