# Supplementary material for: Validation of the Bangla PHQ-4 in rural and urban community samples from Bangladesh
Source: PLoS One. 2026 Jun 26;21(6):e0352459. doi: 10.1371/journal.pone.0352459 (PMC13308809; doi:10.1371/journal.pone.0352459)
Supplement: Attachment — Submitted filename: Response_to_Reviewers_auresp_2.docx [file pone.0352459.s003.docx]

**Response to Review Comments to the Author**

Reviewer #1: Dear authors,
Thank you for your efforts in revising the manuscript. I still have two comments:

1- Criterion-related validity should be added to strengthen the psychometric properties. I understand that this paper is part of a big project but you can still choose one or two scales to correlate the PHQ-4 scores with.

***Response****:*

Based on your suggestion, we have included correlation between the PHQ-4 and K-6, which will add to the construct validity of the PHQ-4. However, please note that we did not use any gold standard (criterion) measure for assessing anxiety or depression. Thus, we cannot claim the criterion validity of the PHQ-4.

We have also made the relevant changes in connection to the inclusion of K6 results. (see line 32, line 140-146, line 246, and line 309)

2- Can you please tell me again why you chose to do the ROC curve and set new cutoff values for this scale and not use the original cutoff values set by the original authors?

***Response****:*

We used the ROC curve for assessing diagnostic performance and sensitivity-specificity of the scale, which was necessary for identifying a suitable cutoff value of the PHQ-4 for Bangladesh. Please note that we are not setting NEW cutoff value for the PHQ-4 in general, we are merely assessing cutoff value for the Bangla PHQ-4 to be used in Bangladesh. As we have mentioned in our earlier reply, “*cutoff value may differ in different cultural context, we had to assess diagnostic performance and cut-off value for the Bangla translated PHQ-4 before the cut-off value can be used in Bangladesh. It should be noted that, this is going to be the first published study with cutoff value of PHQ-4 for Bangladeshi sample.”*

*Please also note that it is a good practice to assess country/context specific cutoff values for the screening tools. Evidence of different cutoff scores of SRQ-20 for different countries can be found at* World Health Organization, (1994) (See Table 5, page 18; available at <https://iris.who.int/bitstream/handle/10665/61113/WHO_MNH_PSF_94.8.pdf?sequence=1%26isAllowed=y> ).

Reviewer #2: You can publish. Be aware you may report p-values with the full number, not just p-value<.05 or p<.01

***Response****:*

Thank you for this suggestion. We understand that reporting full p-values is useful. However, to retain ease of reading, we preferred to use p-values <.05 or <.01, as is common practice.
