# Supplementary material for: Validation of the Bangla PHQ-4 in rural and urban community samples from Bangladesh
Source: PLoS One. 2026 Jun 26;21(6):e0352459. doi: 10.1371/journal.pone.0352459 (PMC13308809; doi:10.1371/journal.pone.0352459)
Supplement: Peer review history

# Peer review: Decision Letter 0

**DOI:** 10.1371/journal.pone.0352459.r001

**Authors:** Flávia Osório

31 Mar 2026

PONE-D-25-67140

Validation of the Bangla PHQ-4 in Rural and Urban Community Samples from Bangladesh

PLOS One

Dear Dr.  Mozumder,

Thank you for submitting your manuscript to PLOS ONE. After careful consideration, we feel that it has merit but does not fully meet PLOS ONE’s publication criteria as it currently stands. Therefore, we invite you to submit a revised version of the manuscript that addresses the points raised during the review process.

Please submit your revised manuscript by May 15 2026 11:59PM. If you will need more time than this to complete your revisions, please reply to this message or contact the journal office at plosone@plos.org. When you're ready to submit your revision, log on to https://www.editorialmanager.com/pone/ and select the 'Submissions Needing Revision' folder to locate your manuscript file.

Please include the following items when submitting your revised manuscript:

- A letter that responds to each point raised by the academic editor and reviewer(s). You should upload this letter as a separate file labeled 'Response to Reviewers'.
- A marked-up copy of your manuscript that highlights changes made to the original version. You should upload this as a separate file labeled 'Revised Manuscript with Track Changes'.
- An unmarked version of your revised paper without tracked changes. You should upload this as a separate file labeled 'Manuscript'.

If you would like to make changes to your financial disclosure, please include your updated statement in your cover letter. Guidelines for resubmitting your figure files are available below the reviewer comments at the end of this letter.

If applicable, we recommend that you deposit your laboratory protocols in protocols.io to enhance the reproducibility of your results. Protocols.io assigns your protocol its own identifier (DOI) so that it can be cited independently in the future. For instructions see: https://journals.plos.org/plosone/s/submission-guidelines#loc-laboratory-protocols. Additionally, PLOS ONE offers an option for publishing peer-reviewed Lab Protocol articles, which describe protocols hosted on protocols.io. Read more information on sharing protocols at https://plos.org/protocols?utm_medium=editorial-email&utm_source=authorletters&utm_campaign=protocols.

As the corresponding author, your ORCID iD is verified in the submission system and will appear in the published article. PLOS supports the use of ORCID, and we encourage all coauthors to register for an ORCID iD and use it as well. Please encourage your coauthors to verify their ORCID iD within the submission system before final acceptance, as unverified ORCID iDs will not appear in the published article. *Only*  the individual author can complete the verification step; PLOS staff *cannot*  verify ORCID iDs on behalf of authors.

We look forward to receiving your revised manuscript.

Kind regards,

Flávia L. Osório, PhD

Academic Editor

PLOS One

Journal Requirements:

When submitting your revision, we need you to address these additional requirements.

1.Please ensure that your manuscript meets PLOS ONE's style requirements, including those for file naming. The PLOS ONE style templates can be found at

https://journals.plos.org/plosone/s/file?id=wjVg/PLOSOne_formatting_sample_main_body.pdf and

https://journals.plos.org/plosone/s/file?id=ba62/PLOSOne_formatting_sample_title_authors_affiliations.pdf

2. In the ethics statement in the Methods, you have specified that verbal consent was obtained. Please provide additional details regarding how this consent was documented and witnessed, and state whether this was approved by the IRB.

3. We note that the grant information you provided in the ‘Funding Information’ and ‘Financial Disclosure’ sections do not match.

When you resubmit, please ensure that you provide the correct grant numbers for the awards you received for your study in the ‘Funding Information’ section.

4. Thank you for stating the following financial disclosure:

“This research received financial and logistic support BRAC Health Programme (BHP), a non-government organization in Bangladesh, for data collection. However, no funding was received for preparing the manuscript or for publication of the study.”

Please state what role the funders took in the study.  If the funders had no role, please state: "The funders had no role in study design, data collection and analysis, decision to publish, or preparation of the manuscript."

If this statement is not correct you must amend it as needed.

Please include this amended Role of Funder statement in your cover letter; we will change the online submission form on your behalf.

5. Please note that your Data Availability Statement is currently missing the repository name and/or the DOI/accession number of each dataset OR a direct link to access each database. If your manuscript is accepted for publication, you will be asked to provide these details on a very short timeline. We therefore suggest that you provide this information now, though we will not hold up the peer review process if you are unable.

6. If the reviewer comments include a recommendation to cite specific previously published works, please review and evaluate these publications to determine whether they are relevant and should be cited. There is no requirement to cite these works unless the editor has indicated otherwise.

[Note: HTML markup is below. Please do not edit.]

Reviewer's Responses to Questions

**Comments to the Author**

1. Is the manuscript technically sound, and do the data support the conclusions?

The manuscript must describe a technically sound piece of scientific research with data that supports the conclusions. Experiments must have been conducted rigorously, with appropriate controls, replication, and sample sizes. The conclusions must be drawn appropriately based on the data presented.

Reviewer #1: Yes

Reviewer #2: Yes

**********

2. Has the statistical analysis been performed appropriately and rigorously?

Reviewer #1: No

Reviewer #2: Yes

**********

3. Have the authors made all data underlying the findings in their manuscript fully available?

The PLOS Data policy requires authors to make all data underlying the findings described in their manuscript fully available without restriction, with rare exception (please refer to the Data Availability Statement in the manuscript PDF file). The data should be provided as part of the manuscript or its supporting information, or deposited to a public repository. For example, in addition to summary statistics, the data points behind means, medians and variance measures should be available. If there are restrictions on publicly sharing data—e.g. participant privacy or use of data from a third party—those must be specified.

Reviewer #1: No

Reviewer #2: Yes

**********

4. Is the manuscript presented in an intelligible fashion and written in standard English?

PLOS ONE does not copyedit accepted manuscripts, so the language in submitted articles must be clear, correct, and unambiguous. Any typographical or grammatical errors should be corrected at revision, so please note any specific errors here.

Reviewer #1: No

Reviewer #2: Yes

**********

5. Review Comments to the Author

Please use the space provided to explain your answers to the questions above. You may also include additional comments for the author, including concerns about dual publication, research ethics, or publication ethics. (Please upload your review as an attachment if it exceeds 20,000 characters)

Reviewer #1: Dear authors,

Thank you for your efforts. The idea is good and necessary for the country and native language, however, the paper needs a lot of work. Here are my comments:

-The paper needs to be corrected by a professional English native speaker. The sentences structure is weak.

-The introduction needs to be reshuffled as follows:

start with a general introduction about mental health status in the world and in the country.

state all scales that are used to assess depression and anxiety and state which ones are validated in Bangladesh

then you should mention why you want to validate the PHQ-4 scale.

State the languages PHQ-4 is validated in

Rationale for validating the scale in Bangladesh

Objectives and hypotheses

Regarding the methods:

-Table 1 contains a low number of participants for certain categories. These categories should be merged together to have a decent number for the analysis.

-The estimation method should be WLSMV since the data is ordinal. Can the authors explain why they used the maximum likelihood? Otherwise, please redo the analysis using WLSMV.

-The fit indices should be obtained from the total sample.

-Measurement invariance should be done by gender, by region of living, education level, etc (as deemed necessary by the authors). The stated results are not fully accurate.

-The authors did not use other scales to assess convergent, divergent, or criterion validity?

-The PHQ-4 scale has an already established cutoff values. Why did the authors do a ROC curve analysis? Please explain.

For the discussion, the results of the current study should be compared to the ones from other countries (fit indices, alpha and omega values, measurement invariance, etc.)

-Authors should add a clinical implications paragraph to state how their results would benefit other healthcare professionals.

-Did the authors do a test-retest?

I hope you find these comments helpful. Good luck and Happy New Year 2026.

Reviewer #2: First of all, thank you very much for the opportunity to review this manuscript. Overall, the paper is well structured, comprehensive, and addresses a highly valuable topic: the measurement of instruments in psychology. This area has wide-ranging applications and represents a meaningful contribution to scientific knowledge.

With the aim of strengthening the manuscript, I would like to offer the following comments and suggestions:

Please consider citing the following article: https://www.nature.com/articles/s41598-023-41560-0

Line 91: Please specify the initial and final sample sizes, as well as any missing data. The current wording in this line raises some uncertainty.

Table 1: Please indicate (e.g. a chi-square test or t-test) whether there are statistically significant differences in sociodemographic characteristics such as sex and age according to place of origin.

Line 114: Please verify whether the reference cited is correct.

The sampling procedure raises several questions. Was a standardised protocol used when interacting with participants? If so, could you provide or publish the script or guidelines used? A more detailed description of the recruitment and approach would be beneficial.

Where were the interviews conducted (e.g., in the street)? How long did they last? It is understood that four instruments were administered: PHQ-4, SRQ-20, a self-rated mental health measure, and informed consent. It is also assumed that participants were informed about the study, its duration, and the consequences of declining or withdrawing, as well as whether they had previously participated in the study with another interviewer. Please clarify these aspects.

A subsection on statistical analysis is missing within the Methods section. I recommend incorporating the content currently presented in lines 147–155 into this subsection, with the Results section beginning at line 156.

Line 163 appears to describe methodological aspects. Please move this content to the statistical analysis subsection.

Similarly, the content in line 169 relates to statistical methodology and should be included in the appropriate subsection.

The term “MH” (presumably referring to mental health) is not defined in the manuscript. Please clarify its meaning.

Content in line 179 should be relocated to the Methods section.

Within the Methods section, it is not sufficiently clear that, following the interviews, the interviewers conducted a diagnostic assessment based on participants’ responses, classifying cases as either positive or negative for impaired mental health. This clinical classification appears to have been subsequently used for the AUC analysis. Please clarify this process.

Table 2: Please omit leading zeros before decimal points.

Please apply the “closest top-left” method to estimate the optimal cut-off point (alternatively, Youden’s index could also be considered). At present, the justification for selecting a cut-off point of 6 as optimal is not sufficiently supported.

Lines 217, 218, and 230: Please report values to three decimal places.

**********

6. PLOS authors have the option to publish the peer review history of their article (what does this mean?). If published, this will include your full peer review and any attached files.

If you choose “no”, your identity will remain anonymous but your review may still be made public.

**Do you want your identity to be public for this peer review?**  For information about this choice, including consent withdrawal, please see our Privacy Policy.

Reviewer #1: No

Reviewer #2: No

**********

[NOTE: If reviewer comments were submitted as an attachment file, they will be attached to this email and accessible via the submission site. Please log into your account, locate the manuscript record, and check for the action link "View Attachments". If this link does not appear, there are no attachment files.]

To ensure your figures meet our technical requirements, please review our figure guidelines: https://journals.plos.org/plosone/s/figures

You may also use PLOS’s free figure tool, NAAS, to help you prepare publication quality figures: https://journals.plos.org/plosone/s/figures#loc-tools-for-figure-preparation.

NAAS will assess whether your figures meet our technical requirements by comparing each figure against our figure specifications.

# Author comment: Author response to Decision Letter 1

**DOI:** 10.1371/journal.pone.0352459.r002

27 May 2026

Response to Review Comments to the Author

Reviewer #1:

Dear authors, Thank you for your efforts. The idea is good and necessary for the country and native language, however, the paper needs a lot of work. Here are my comments:

Thank you for your support. We truly appreciate the contribution of the reviewers in gatekeeping knowledge. We have tried our best to address your comments.

-The paper needs to be corrected by a professional English native speaker. The sentences structure is weak.

Response:

We understand that reading English-written work by a non-native speaker can be uncomfortable, and we sincerely apologise for that. Unfortunately, none of the team members was a native speaker. After your comment, we invited a British-born graduate student, a British-born post-graduate student, and a British-born Medical Doctor to review the work, and all of them cleared it for any lack of understanding or major grammatical errors. Nonetheless, we have revised some linguistic and grammatical issues as well as typos to improve the readability of the work.

-The introduction needs to be reshuffled as follows:

start with a general introduction about mental health status in the world and in the country.

state all scales that are used to assess depression and anxiety and state which ones are validated in Bangladesh

then you should mention why you want to validate the PHQ-4 scale.

State the languages PHQ-4 is validated in

Rationale for validating the scale in Bangladesh

Objectives and hypotheses

Response:

We preferred to present an opening paragraph at the beginning to introduce the idea to the readers. However, from the second paragraph onward, we gradually funnel down toward the objective (the team preferred to phrase this as ‘aim’ instead of ‘objective’). Moreover, we have made a few revisions as per your suggestion. Details on the locally available tools for assessing depression and anxiety have been added (line 64-73). We have also added languages in which the PHQ-4 has been validated (line 82-85). Additionally, we have added details on validation findings from published literature (line 108-110; line 112-114).

Regarding the methods:

-Table 1 contains a low number of participants for certain categories. These categories should be merged together to have a decent number for the analysis.

Response:

We have merged several subcategories under ‘marital status’ and ‘source of income’ as per your suggestion. We have also added a new categorisation of age. (Table 1)

-The estimation method should be WLSMV since the data is ordinal. Can the authors explain why they used the maximum likelihood? Otherwise, please redo the analysis using WLSMV.

Response:

Thank you for this extremely useful suggestion. We have now re-analysed the data with the estimator specified as WLSMV, and have revised the result section accordingly. (line 183-184; line 218-219)

-The fit indices should be obtained from the total sample.

Response:

As per your suggestion, we have kept only the fit indices for the total sample and described findings on fit indices in the text, removing the table. (line 218-219).

-Measurement invariance should be done by gender, by region of living, education level, etc (as deemed necessary by the authors). The stated results are not fully accurate.

Response:

We have carried out measurement invariance tests for sex, age, and mental health state and added the findings as Table 2. (line 225-232)

-The authors did not use other scales to assess convergent, divergent, or criterion validity?

Response:

We already have a plan to conduct a larger study to test the construct validity (convergent, divergent), and test-retest reliability of the tool. However, as this is an already developed scale with proven criterion validity, we did not plan to conduct testing for criterion validity.

-The PHQ-4 scale has an already established cutoff values. Why did the authors do a ROC curve analysis? Please explain.

Response:

As the cutoff value may differ in different cultural contexts, we had to assess diagnostic performance and cut-off value for the Bangla translated PHQ-4 before the cutoff value can be used in Bangladesh. It should be noted that this is going to be the first published study with cutoff value of PHQ-4 for the Bangladeshi sample.

For the discussion, the results of the current study should be compared to the ones from other countries (fit indices, alpha and omega values, measurement invariance, etc.)

Response:

As per your suggestion, we have added a comparative discussion on the psychometric properties of the tool. (line 276- 286, line 290-293; and also line 108-110; line 112-114)

-Authors should add a clinical implications paragraph to state how their results would benefit other healthcare professionals.

Response:

Thank you for this suggestion. We have added a brief paragraph addressing the implications of the findings. (line 324-332)

-Did the authors do a test-retest?

Response:

We have not done test-retest of the tool. We are planning to conduct a larger study to test the construct validity and test-retest reliability of the tool.

I hope you find these comments helpful. Good luck and Happy New Year 2026.

Thank you for spending time reviewing this article. These comments were extremely useful for our team, not only in improving the current article but for our upcoming work as well.

Reviewer #2:

First of all, thank you very much for the opportunity to review this manuscript. Overall, the paper is well structured, comprehensive, and addresses a highly valuable topic: the measurement of instruments in psychology. This area has wide-ranging applications and represents a meaningful contribution to scientific knowledge.

With the aim of strengthening the manuscript, I would like to offer the following comments and suggestions:

Thank you for your support. We truly appreciate the contribution of the reviewers in gatekeeping knowledge.

Please consider citing the following article: https://www.nature.com/articles/s41598-023-41560-0

Response:

We have already cited similar and more recent study findings. We were not sure how citing this would add value to the current article. Therefore, we did not consider citing the suggested article.

Line 91: Please specify the initial and final sample sizes, as well as any missing data. The current wording in this line raises some uncertainty.

Response:

Thank you for this useful feedback. We have the total number of participants and added the necessary details on missing value. (line 103, line 106-111)

Table 1: Please indicate (e.g. a chi-square test or t-test) whether there are statistically significant differences in sociodemographic characteristics such as sex and age according to place of origin.

Response:

We have added the analysis in the findings section. (line 205-212.)

Line 114: Please verify whether the reference cited is correct.

Response:

Thank you for notifying this. We have changed this citation. (line 133)

The sampling procedure raises several questions. Was a standardised protocol used when interacting with participants? If so, could you provide or publish the script or guidelines used? A more detailed description of the recruitment and approach would be beneficial.

Where were the interviews conducted (e.g., in the street)? How long did they last? It is understood that four instruments were administered: PHQ-4, SRQ-20, a self-rated mental health measure, and informed consent. It is also assumed that participants were informed about the study, its duration, and the consequences of declining or withdrawing, as well as whether they had previously participated in the study with another interviewer. Please clarify these aspects.

Response:

Thank you for these very useful comments. We have added some text in the procedure section to improve clarity of understanding as per your comments. (line 154-158, line 161-165, line 167-169, line 171-172, line 174-179)

A subsection on statistical analysis is missing within the Methods section. I recommend incorporating the content currently presented in lines 147–155 into this subsection, with the Results section beginning at line 156.

Line 163 appears to describe methodological aspects. Please move this content to the statistical analysis subsection.

Similarly, the content in line 169 relates to statistical methodology and should be included in the appropriate subsection.

Content in line 179 should be relocated to the Methods section.

Response:

We thought that describing the data analysis method in line with the result would provide the readers with better connectivity. However, we agree with your suggestions and have added a data analysis section incorporating the indicated sentences along with a few connecting sentences. (line 182- 203)

The term “MH” (presumably referring to mental health) is not defined in the manuscript. Please clarify its meaning.

Response:

Thank you for notifying this. Now we have added this to the text as well as under the table. (line # 119, 202-203, 241)

Within the Methods section, it is not sufficiently clear that, following the interviews, the interviewers conducted a diagnostic assessment based on participants’ responses, classifying cases as either positive or negative for impaired mental health. This clinical classification appears to have been subsequently used for the AUC analysis. Please clarify this process.

Response:

To explore the presence of mental health conditions, the interviewer conducted an additional assessment based on the responses provided by the participants. However, as these interviews were not targeted to diagnose a specific disorder, the interviewers did not use SCID or other clinical diagnosis approaches for identifying a specific clinical disorder. As per your comment, details have been added to the instrument section. (line 144-148)

Table 2: Please omit leading zeros before decimal points.

Response:

Thank you for noticing this. Based on the comments of the other reviewer, we have removed the older Table 2 and replaced it with a new one.

As per your comments, we have checked the manuscript and have omitted ‘0’s before the decimal point for p-values, SRMR values, alpha value and omega value. However, as per APA 7th recommendations, we have kept the ‘0’s before the decimal point intact for some other values which can go above ‘1’ .

Please apply the “closest top-left” method to estimate the optimal cut-off point (alternatively, Youden’s index could also be considered). At present, the justification for selecting a cut-off point of 6 as optimal is not sufficiently supported.

Response:

We did not use Youden’s index or other established methods, as these indicate a cutoff with disproportionate sensitivity and specificity (77 and 90). Instead, we used a “least difference method” considering the usefulness of the tool in both directions. In this case, the cutoff 6 gave a more balanced estimate of sensitivity and specificity (82 and 83). However, as we have given the sensitivity and specificity table in the article, any user can choose a suitable cutoff value in accordance with their purpose.

Thanks to your comments, we have added these details to the manuscript for the readers. (line 257-261, line 315-318)

Lines 217, 218, and 230: Please report values to three decimal places.

Response:

Revised as per the suggestion

Thank you for spending time reviewing this article. These comments were useful for our team, not only in improving the current article but for our upcoming work as well.

Attachment Submitted filename: Response to Reviewers.docx

# Peer review: Decision Letter 1

**DOI:** 10.1371/journal.pone.0352459.r003

**Authors:** Flávia Osório, Flávia Osório

3 Jun 2026

PONE-D-25-67140R1

Validation of the Bangla PHQ-4 in Rural and Urban Community Samples from Bangladesh

PLOS One

Dear Dr.  Mozumder,

Thank you for submitting your manuscript to PLOS ONE. After careful consideration, we feel that it has merit but does not fully meet PLOS ONE’s publication criteria as it currently stands. Therefore, we invite you to submit a revised version of the manuscript that addresses the points raised during the review process.

Please submit your revised manuscript by Jul 18 2026 11:59PM. If you will need more time than this to complete your revisions, please reply to this message or contact the journal office at plosone@plos.org. When you're ready to submit your revision, log on to https://www.editorialmanager.com/pone/ and select the 'Submissions Needing Revision' folder to locate your manuscript file.

Please include the following items when submitting your revised manuscript:

- A letter that responds to each point raised by the academic editor and reviewer(s). You should upload this letter as a separate file labeled 'Response to Reviewers'.
- A marked-up copy of your manuscript that highlights changes made to the original version. You should upload this as a separate file labeled 'Revised Manuscript with Track Changes'.
- An unmarked version of your revised paper without tracked changes. You should upload this as a separate file labeled 'Manuscript'.

If you would like to make changes to your financial disclosure, please include your updated statement in your cover letter. Guidelines for resubmitting your figure files are available below the reviewer comments at the end of this letter.

If applicable, we recommend that you deposit your laboratory protocols in protocols.io to enhance the reproducibility of your results. Protocols.io assigns your protocol its own identifier (DOI) so that it can be cited independently in the future. For instructions see: https://journals.plos.org/plosone/s/submission-guidelines#loc-laboratory-protocols. Additionally, PLOS ONE offers an option for publishing peer-reviewed Lab Protocol articles, which describe protocols hosted on protocols.io. Read more information on sharing protocols at https://plos.org/protocols?utm_medium=editorial-email&utm_source=authorletters&utm_campaign=protocols.

As the corresponding author, your ORCID iD is verified in the submission system and will appear in the published article. PLOS supports the use of ORCID, and we encourage all coauthors to register for an ORCID iD and use it as well. Please encourage your coauthors to verify their ORCID iD within the submission system before final acceptance, as unverified ORCID iDs will not appear in the published article. *Only*  the individual author can complete the verification step; PLOS staff *cannot*  verify ORCID iDs on behalf of authors.

We look forward to receiving your revised manuscript.

Kind regards,

Flávia L. Osório, PhD

Academic Editor

PLOS One

Journal Requirements:

1. If the reviewer comments include a recommendation to cite specific previously published works, please review and evaluate these publications to determine whether they are relevant and should be cited. There is no requirement to cite these works unless the editor has indicated otherwise.

2. Please review your reference list to ensure that it is complete and correct. If you have cited papers that have been retracted, please include the rationale for doing so in the manuscript text, or remove these references and replace them with relevant current references. Any changes to the reference list should be mentioned in the rebuttal letter that accompanies your revised manuscript. If you need to cite a retracted article, indicate the article’s retracted status in the References list and also include a citation and full reference for the retraction notice.

[Note: HTML markup is below. Please do not edit.]

Reviewer's Responses to Questions

**Comments to the Author**

1. If the authors have adequately addressed your comments raised in a previous round of review and you feel that this manuscript is now acceptable for publication, you may indicate that here to bypass the “Comments to the Author” section, enter your conflict of interest statement in the “Confidential to Editor” section, and submit your "Accept" recommendation.

Reviewer #1: All comments have been addressed

Reviewer #2: All comments have been addressed

**********

2. Is the manuscript technically sound, and do the data support the conclusions?

The manuscript must describe a technically sound piece of scientific research with data that supports the conclusions. Experiments must have been conducted rigorously, with appropriate controls, replication, and sample sizes. The conclusions must be drawn appropriately based on the data presented.

Reviewer #1: Yes

Reviewer #2: Yes

**********

3. Has the statistical analysis been performed appropriately and rigorously?

Reviewer #1: Yes

Reviewer #2: Yes

**********

4. Have the authors made all data underlying the findings in their manuscript fully available?

The PLOS Data policy requires authors to make all data underlying the findings described in their manuscript fully available without restriction, with rare exception (please refer to the Data Availability Statement in the manuscript PDF file). The data should be provided as part of the manuscript or its supporting information, or deposited to a public repository. For example, in addition to summary statistics, the data points behind means, medians and variance measures should be available. If there are restrictions on publicly sharing data—e.g. participant privacy or use of data from a third party—those must be specified.

Reviewer #1: No

Reviewer #2: Yes

**********

5. Is the manuscript presented in an intelligible fashion and written in standard English?

PLOS ONE does not copyedit accepted manuscripts, so the language in submitted articles must be clear, correct, and unambiguous. Any typographical or grammatical errors should be corrected at revision, so please note any specific errors here.

Reviewer #1: Yes

Reviewer #2: Yes

**********

6. Review Comments to the Author

Please use the space provided to explain your answers to the questions above. You may also include additional comments for the author, including concerns about dual publication, research ethics, or publication ethics. (Please upload your review as an attachment if it exceeds 20,000 characters)

Reviewer #1: Dear authors,

Thank you for your efforts in revising the manuscript. I still have two comments:

1- Criterion-related validity should be added to strengthen the psychometric properties. I understand that this paper is part of a big project but you can still choose one or two scales to correlate the PHQ-4 scores with.

2- Can you please tell me again why you chose to do the ROC curve and set new cutoff values for this scale and not use the original cutoff values set by the original authors?

Good luck with your future projects.

Reviewer #2: You can publish. Be aware you may report p values with the full number, not just p-value<.05 or p<.01

**********

7. PLOS authors have the option to publish the peer review history of their article (what does this mean?). If published, this will include your full peer review and any attached files.

If you choose “no”, your identity will remain anonymous but your review may still be made public.

**Do you want your identity to be public for this peer review?**  For information about this choice, including consent withdrawal, please see our Privacy Policy.

Reviewer #1: No

Reviewer #2: **Yes:** Armando González-Sánchez

**********

[NOTE: If reviewer comments were submitted as an attachment file, they will be attached to this email and accessible via the submission site. Please log into your account, locate the manuscript record, and check for the action link "View Attachments". If this link does not appear, there are no attachment files.]

To ensure your figures meet our technical requirements, please review our figure guidelines: https://journals.plos.org/plosone/s/figures

You may also use PLOS’s free figure tool, NAAS, to help you prepare publication quality figures: https://journals.plos.org/plosone/s/figures#loc-tools-for-figure-preparation.

NAAS will assess whether your figures meet our technical requirements by comparing each figure against our figure specifications.

# Author comment: Author response to Decision Letter 2

**DOI:** 10.1371/journal.pone.0352459.r004

5 Jun 2026

Reviewer #1: Dear authors,

Thank you for your efforts in revising the manuscript. I still have two comments:

1- Criterion-related validity should be added to strengthen the psychometric properties. I understand that this paper is part of a big project but you can still choose one or two scales to correlate the PHQ-4 scores with.

Response:

Based on your suggestion, we have included correlation between the PHQ-4 and K-6, which will add to the construct validity of the PHQ-4. However, please note that we did not use any gold standard (criterion) measure for assessing anxiety or depression. Thus, we cannot claim the criterion validity of the PHQ-4.

We have also made the relevant changes in connection to the inclusion of K6 results. (see line 32, line 140-146, line 246, and line 309)

2- Can you please tell me again why you chose to do the ROC curve and set new cutoff values for this scale and not use the original cutoff values set by the original authors?

Response:

We used the ROC curve for assessing diagnostic performance and sensitivity-specificity of the scale, which was necessary for identifying a suitable cutoff value of the PHQ-4 for Bangladesh. Please note that we are not setting NEW cutoff value for the PHQ-4 in general, we are merely assessing cutoff value for the Bangla PHQ-4 to be used in Bangladesh. As we have mentioned in our earlier reply, “cutoff value may differ in different cultural context, we had to assess diagnostic performance and cut-off value for the Bangla translated PHQ-4 before the cut-off value can be used in Bangladesh. It should be noted that, this is going to be the first published study with cutoff value of PHQ-4 for Bangladeshi sample.”

Please also note that it is a good practice to assess country/context specific cutoff values for the screening tools. Evidence of different cutoff scores of SRQ-20 for different countries can be found at World Health Organization, (1994) (See Table 5, page 18; available at https://iris.who.int/bitstream/handle/10665/61113/WHO_MNH_PSF_94.8.pdf?sequence=1%26isAllowed=y ).

Reviewer #2: You can publish. Be aware you may report p-values with the full number, not just p-value<.05 or p<.01

Response:

Thank you for this suggestion. We understand that reporting full p-values is useful. However, to retain ease of reading, we preferred to use p-values <.05 or <.01, as is common practice.

Attachment Submitted filename: Response_to_Reviewers_auresp_2.docx

# Peer review: Decision Letter 2

**DOI:** 10.1371/journal.pone.0352459.r005

**Authors:** Flávia Osório, Flávia Osório, Flávia Osório

10 Jun 2026

<p>Validation of the Bangla PHQ-4 in Rural and Urban Community Samples from Bangladesh

PONE-D-25-67140R2

Dear Dr. Mozumder,

We’re pleased to inform you that your manuscript has been judged scientifically suitable for publication and will be formally accepted for publication once it meets all outstanding technical requirements.

Within one week, you’ll receive an e-mail detailing the required amendments. When these have been addressed, you’ll receive a formal acceptance letter and your manuscript will be scheduled for publication.

An invoice will be generated when your article is formally accepted. Please note, if your institution has a publishing partnership with PLOS and your article meets the relevant criteria, all or part of your publication costs will be covered. Please make sure your user information is up-to-date by logging into Editorial Manager at Editorial Manager® and clicking the ‘Update My Information' link at the top of the page. For questions related to billing, please contact billing support.

If your institution or institutions have a press office, please notify them about your upcoming paper to help maximize its impact. If they’ll be preparing press materials, please inform our press team as soon as possible -- no later than 48 hours after receiving the formal acceptance. Your manuscript will remain under strict press embargo until 2 pm Eastern Time on the date of publication. For more information, please contact onepress@plos.org.

Kind regards,

Flávia L. Osório, PhD

Academic Editor

PLOS One

Additional Editor Comments (optional):

Reviewers' comments:

Reviewer's Responses to Questions

**Comments to the Author**

1. If the authors have adequately addressed your comments raised in a previous round of review and you feel that this manuscript is now acceptable for publication, you may indicate that here to bypass the “Comments to the Author” section, enter your conflict of interest statement in the “Confidential to Editor” section, and submit your "Accept" recommendation.

Reviewer #1: All comments have been addressed

**********

2. Is the manuscript technically sound, and do the data support the conclusions?

The manuscript must describe a technically sound piece of scientific research with data that supports the conclusions. Experiments must have been conducted rigorously, with appropriate controls, replication, and sample sizes. The conclusions must be drawn appropriately based on the data presented.

Reviewer #1: Yes

**********

3. Has the statistical analysis been performed appropriately and rigorously?

Reviewer #1: Yes

**********

4. Have the authors made all data underlying the findings in their manuscript fully available?

The PLOS Data policy requires authors to make all data underlying the findings described in their manuscript fully available without restriction, with rare exception (please refer to the Data Availability Statement in the manuscript PDF file). The data should be provided as part of the manuscript or its supporting information, or deposited to a public repository. For example, in addition to summary statistics, the data points behind means, medians and variance measures should be available. If there are restrictions on publicly sharing data—e.g. participant privacy or use of data from a third party—those must be specified.

Reviewer #1: No

**********

5. Is the manuscript presented in an intelligible fashion and written in standard English?

PLOS ONE does not copyedit accepted manuscripts, so the language in submitted articles must be clear, correct, and unambiguous. Any typographical or grammatical errors should be corrected at revision, so please note any specific errors here.

Reviewer #1: Yes

**********

6. Review Comments to the Author

Please use the space provided to explain your answers to the questions above. You may also include additional comments for the author, including concerns about dual publication, research ethics, or publication ethics. (Please upload your review as an attachment if it exceeds 20,000 characters)

Reviewer #1: Dear authors,

Thank you for your efforts in addressing the comments.

The paper is now publishable in its current form.

Best of luck for future projects.

**********

7. PLOS authors have the option to publish the peer review history of their article (what does this mean?). If published, this will include your full peer review and any attached files.

If you choose “no”, your identity will remain anonymous but your review may still be made public.

**Do you want your identity to be public for this peer review?**  For information about this choice, including consent withdrawal, please see our Privacy Policy.

Reviewer #1: No

**********

# Editor report: Acceptance letter

**DOI:** 10.1371/journal.pone.0352459.r006

**Authors:** Flávia Osório, Flávia Osório, Flávia Osório

PONE-D-25-67140R2

PLOS One

Dear Dr. Mozumder,

I'm pleased to inform you that your manuscript has been deemed suitable for publication in PLOS One. Congratulations! Your manuscript is now being handed over to our production team.

At this stage, our production department will prepare your paper for publication. This includes ensuring the following:

* All references, tables, and figures are properly cited

* All relevant supporting information is included in the manuscript submission,

* There are no issues that prevent the paper from being properly typeset

You will receive further instructions from the production team, including instructions on how to review your proof when it is ready. Please keep in mind that we are working through a large volume of accepted articles, so please give us a few days to review your paper and let you know the next and final steps.

Lastly, if your institution or institutions have a press office, please let them know about your upcoming paper now to help maximize its impact. If they'll be preparing press materials, please inform our press team within the next 48 hours. Your manuscript will remain under strict press embargo until 2 pm Eastern Time on the date of publication. For more information, please contact onepress@plos.org.

You will receive an invoice from PLOS for your publication fee after your manuscript has reached the completed accept phase. If you receive an email requesting payment before acceptance or for any other service, this may be a phishing scheme. Learn how to identify phishing emails and protect your accounts at https://explore.plos.org/phishing.

If we can help with anything else, please email us at customercare@plos.org.

Thank you for submitting your work to PLOS ONE and supporting open access.

Kind regards,

PLOS ONE Editorial Office Staff

on behalf of

Dr. Flávia L. Osório

Academic Editor

PLOS One
